# Endoscopic Submucosal Tunnel Dissection for Large Gastric Neoplastic Lesions: A Case-Matched Controlled Study

**DOI:** 10.1155/2018/1419369

**Published:** 2018-03-06

**Authors:** Xiuxue Feng, Enqiang Linghu, Ningli Chai, Zhongsheng Lu, Xiangdong Wang, Ping Tang, Jiangyun Meng, Hong Du, Hongbin Wang

**Affiliations:** Department of Gastroenterology, Chinese PLA General Hospital, Beijing 100853, China

## Abstract

**Aim:**

To evaluate the efficacy and safety of endoscopic submucosal tunnel dissection (ESTD) for resection of large superficial gastric lesions (SGLs).

**Methods:**

The clinicopathological records of patients performed with ESTD or endoscopic submucosal dissection (ESD) for SGLs between January 2012 and January 2014 were retrospectively reviewed. 7 cases undergoing ESTD were enrolled to form the ESTD group. The cases were individually matched at a 1 : 1 ratio to other patients performed with ESD according to lesion location, ulcer or scar findings, resected specimen area, operation time and operators, and the matched cases constituting the ESD group. The treatment outcomes were compared between the two groups.

**Results:**

The mean specimen size was 46 mm. 10 lesions were located in the cardia and 4 lesions in the lesser curvature of the lower gastric body. En bloc resection was achieved for all lesions. The mean ESTD resection time was 69 minutes as against 87.7 minutes for the ESD (*P* = 0.01). The mean resection speed was faster for ESTD than for ESD (18.86 mm^2^/min versus 13.76 mm^2^/min, *P* = 0.03). There were no significant differences regarding the safety and curability during the endoscopic follow-up (mean 27 months).

**Conclusions:**

ESTD is effective and safe for the removal of SGLs and appears to be an optimal option for patients with large SGLs at suitable sites.

## 1. Introduction

The widespread use of gastroscopy and equipment innovations in endoscopic technology has increased the detection rate of superficial gastric lesions (SGLs) [1, 2]. Progress has improved the resectability of endoscopic techniques, thereby sparing patients from potentially major ablative surgery [3]. Meantime, as the acceptance of expanded indications of endoscopic resection, endoscopists have to face an increasing number of patients with large SGLs. Endoscopic submucosal dissection (ESD) has been established as one standard treatment for SGLs, providing a higher en bloc resection rate and more accurate pathological evaluation than endoscopic mucosal resection (EMR) [1, 2, 4]. Although ESD enables en bloc resection regardless of the lesion size, conventional ESD is time-consuming and poses high risk for large lesions. The main influencing factor in ESD operation for large lesions is poor visualization of the submucosal layer due to contraction or curling of the resected mucosa [5–17]. Therefore, how to lift the submucosal layer and dissect large lesions under direct vision becomes a very challenging problem.

Several traction methods for ESD have been investigated to overcome the problem, such as percutaneous traction [5], clip with line [6–8], clip-and-snare [9], external grasping forceps [10], internal traction [11, 12], suture-pulley [13], magnetic anchor [14], double-channel endoscope [15], double-endoscope [16], and robot-assisted method [17]. However, those traction methods need extra devices or equipment and may be invasive or difficult to control the pulley strength and direction or inconvenient to be operated. Therefore, ESD techniques remain to be further improved to establish the most ideal method for large lesions.

With the advent of the submucosal tunneling technique, endoscopic application has been expanded. In this technique, one submucosal tunnel is created to provide a working space for endoscopic interventions, including resection of gastrointestinal neoplastic lesions [3, 18–21] and submucosal tumors [22, 23], myotomy for achalasia [24] and gastroparesis [25], and even to permit safer access to the peritoneal and thoracic cavity for related diagnosis and treatment [26, 27]. Endoscopic submucosal tunnel dissection (ESTD) secures a stable and good view for the dissection through the submucosal tunnel, which facilitates the lateral mucosal stretching, easy insufflation with air, and maintaining the effect of submucosal injection. Previous studies have shown that ESTD is quick and effective in the resection of large esophageal neoplastic lesions [19]. In porcine models, ESTD was proven to be feasible and safe for SGLs and provides a better quality histologic specimen than ESD [3]. Additionally, Choi et al. [18] reported that ESTD was feasible for two cases of ulcerative early gastric cancer. Based on the experience of ESTD for large esophageal neoplastic lesions and upper gastrointestinal submucosal tumors, we attempted ESTD to improve the efficacy and safety of ESD for large SGLs from 2012. The aim of this study was to evaluate the efficacy and safety of ESTD compared with conventional ESD for large SGLs based on a case-matched controlled analysis.

## 2. Methods

### 2.1. Patients

The study was reviewed and approved by the institutional review board of Chinese PLA General Hospital. The medical and endoscopic records of patients performed ESD with or without tunneling method in our institute for SGLs between January 2012 and January 2014 were retrospectively reviewed. To achieve accurate operation time, the patients with more than one lesions resected simultaneously were excluded. There were 8 cases who underwent ESD with the tunneling method, but one was excluded because of one submucosal tumor resected simultaneously in the same location. Then, the other 7 cases were enrolled to form the ESTD group. The cases were individually matched at a 1 : 1 ratio to other patients undergoing conventional ESD according to lesion location, ulcer or scar findings, resected specimen area (±100 mm^2^), operation time (±6 months) and operators, and the matched ones constituting the ESD group. When more than one control patient was matched, the patient with the date of endoscopic operation closest to the corresponding operation was selected. All of the patients signed the informed consent prior to the endoscopic therapy.

### 2.2. ESD and ESTD Procedures

All of the procedures in this study were performed under general anesthesia by 2 experienced operators, who had completed more than 100 ESD cases before January 2012. Magnified narrow-band imaging (M-NBI) and chromoendoscopy (using indigo carmine) were used to determine lesion area before the operation. The endoscopic equipment and accessories used in the operation included a single-accessory channel endoscope (GIF-Q260J; Olympus, Tokyo, Japan) with a transparent cap (D-201-11804; Olympus) attached to the front, a high-frequency generator (ICC-200; ERBE Elektromedizin, Tübingen, Germany), an argon plasma coagulation unit (APC 300; ERBE) for marking, an injection needle (INJ1-A1; Medwork, Höchstadt, Germany), a dual knife (KD-650L; Olympus) for cutting or circumferential incision or dissection, an insulated-tip (IT) knife (KD-611L; Olympus) or a hook knife (KD-620LR; Olympus) for circumferential incision or dissection or bilateral resection, and hot biopsy forceps (FD-410LR; Olympus) for hemostasis. Carbon dioxide insufflation was used during all the procedures. Normal saline with 0.1% methylene blue and 0.5% epinephrine was injected into the submucosal layer to elevate the lesion.

**Figure 1:**
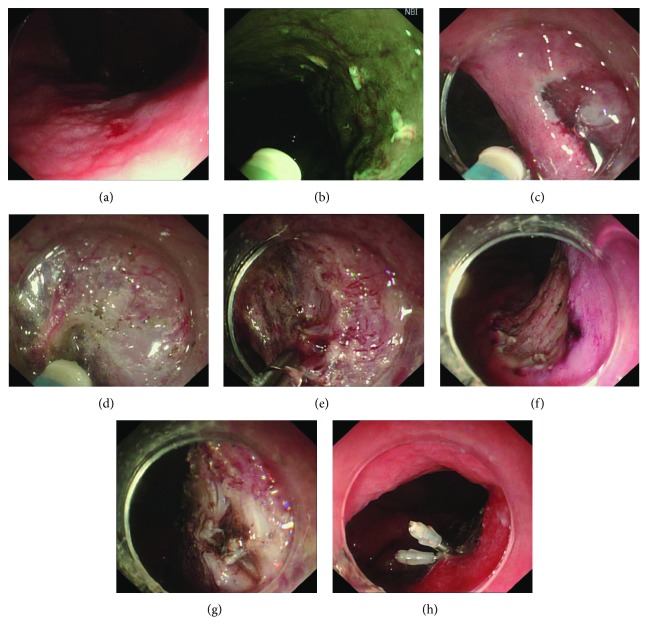
ESD procedure. (a) Lesion under light endoscopy. (b) Marking the margin. (c) Circumferential incision. (d) Submucosal dissection. (e) Hemostasis with hot biopsy forceps. (f) The artificial ulcer after complete removal of the lesion. (g) The muscularis propria damage. (h) The damage was closed with clips to prevent perforation.

**Figure 2:**
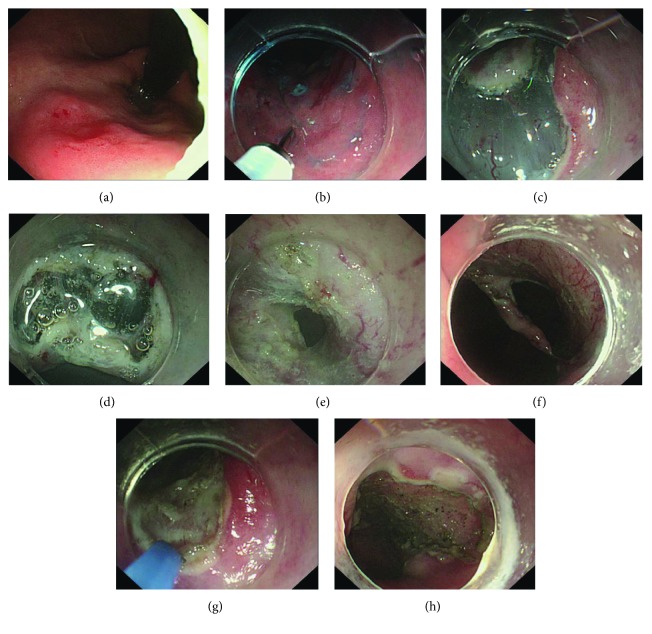
ESTD procedure. (a) Lesion under light endoscopy. (b) Marking the margin followed by submucosal injection. (c) Anal incision. (d) Oral incision. (e) One tunnel was established from oral to anal incision through submucosal dissection. (f) Bilateral resection. (g) Visible vessels were preventatively coagulated with APC. (h) The artificial ulcer after en bloc resection of the lesion.

The ESD procedure was previously described in detail [8], namely, marking, submucosal injection, circumferential incision, and dissection. The comprehensive ESTD procedure was recorded in our published book [28], and the ESTD standard procedure was briefly presented as marking, submucosal injection, anal incision, oral incision, tunnel creation, and bilateral resection. For the lesions in the lower curve of the gastric body, the tunnel was created in retroflex approach from the anal to oral side. The two different procedures are shown in Figures 1 and 2. After complete removal of the lesion, the artificial ulcer was reassessed and visible vessels were routinely coagulated with hemostatic forceps or argon plasma coagulation. The resected specimen was immediately pinned flat to a rubber plate for measurement and imaging and then fixed into formalin for subsequent histopathological evaluation. Then, the specimen was sliced at 2 mm intervals. Each slice was processed for histopathological assessment of histological type, invasion depth, horizontal and vertical margins, and lymphovascular invasion.

### 2.3. Postoperative Treatment

After the operation, the patients were observed closely for complications, such as bleeding, perforation, and infection, and were given immediate treatment when necessary. In the absence of any complications, water intake was permitted on the second day, and the diet of the patients was changed gradually from clear liquid diet to semiliquid diet from the third day. Proton pump inhibitors were prescribed for 2 months and antibiotics for at least 3 days.

### 2.4. Outcomes and Definitions

To evaluate the safety and efficacy of ESTD, the following outcomes were analyzed between the two groups: resection time, size and area of the resected specimen, resection speed, en bloc resection rate, complete resection rate, recurrence rate, and rates of complications, including the muscularis propria (MP) damage, perforation, and postprocedural bleeding. The follow-up data were also analyzed to assess the curability of ESTD.

The macroscopic types and the depth of invasion were classified according to the Paris endoscopic classification of superficial neoplastic lesions [29]. The resection time was defined as the time from the start of cutting to the completion of the resection, including handling the ulcer. The resected specimen was measured directly after resection and imaged; the picture stored in the endoscopic database. The specimen size was defined as the maximum diameter. The specimen area was calculated as follows: area (mm^2^) = major axis (mm) × minor axis (mm) × 3.14/4. The resection speed (mm^2^/min) was calculated as the area of the resected specimen (mm^2^) divided by the resection time (minutes). En bloc resection meant removal of the lesion in one piece. Complete resection was defined as the lesion was removed as one piece with pathologically negative margins. Curative resection was considered when the lesion met the absolute and expanded indications [30]. After removal of the primary lesion, local recurrence was diagnosed when a similar or worse lesion was detected at the primary resection site after at least two negative follow-ups of endoscopic examination. A new lesion detected at a location different from the primary resected lesion within 12 months was defined as synchronous recurrence, and a new lesion detected at more than 12 months was regarded as metachronous recurrence [31]. MP damage meant the coagulation change of MP observed from the artificial ulcer after the procedure. Perforation was diagnosed if the extramural organ or tissue was visualized under endoscopy or if free air was observed on abdominal radiography or computed tomography [8]. Postprocedural bleeding was diagnosed when two of the four following parameters were satisfied after the procedure: (i) hematemesis, melena, or dizziness; (ii) a blood pressure decrease of >20 mmHg or a pulse rate increase of >20 times/min; (iii) decrease in the hemoglobin level of at least 2 g/dL; and (iv) endoscopic confirmation of bleeding from the artificial ulcer by presenting active bleeding, exposed vessels and/or fresh clots that were not seen immediately after the operation or an evident increase in clots in the stomach compared observations during the operation [32].

### 2.5. Statistical Analysis

Quantitative data were presented as mean ± standard deviation (SD). Comparisons between the two groups were assessed using the paired sample *t-*tests for continuous variables and the chi-square or Fisher's exact test for categorical variables. The Wilcoxon signed-rank test was used when equal variances were not assumed. *P* < 0.05 was considered significant for all tests.

## 3. Results

**Figure 3:**
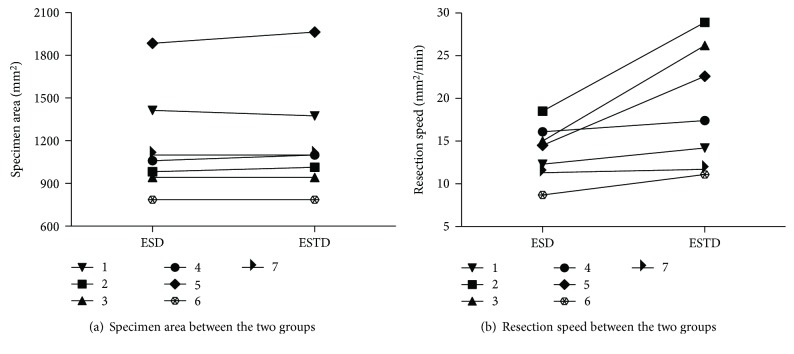
Graph representing the changes of the paired cases. (a) The specimen areas are similar between the two groups. (b) Compared with ESD, ESTD presents faster resection speed in all pairs.

The detailed baseline characteristics and treatment outcomes of the lesions in the ESTD and ESD groups are shown in Table 1. The mean specimen size was 46 mm (range 40–60 mm). 10 lesions were located in the cardia (6 mainly in lesser curvature and 4 mainly in the posterior wall) and 4 lesions in the lesser curvature of the lower gastric body. En bloc resection was achieved for all lesions. No differences in the baseline characteristics of the patients and lesions were found between the groups (*P* > 0.05), including all of the matched factors. As shown in Figure 3(a), the mean specimen area in the two group was similar (1166.29 mm^2^ in ESD versus 1181.99 mm^2^ in ESTD, *P* > 0.05). The mean ESTD resection time was 69 minutes as against 87.7 minutes for the ESD (*P* = 0.0.01). The mean resection speed was faster for ESTD than for ESD (18.86 mm^2^/min versus 13.76 mm^2^/min, *P* = 0.03), and the differences of resection speed were shown in Figure 3(b). No complications were observed in the ESTD group, but one case with MP damage was found in the ESD group and the damage was closed with two clips.

Histopathological evaluation of the resected specimens revealed 3 dysplasia, 4 cancers in the ESTD group, 1 hyperplastic polyp, 1 dysplasia, and 5 cancers in the ESD group. Among the cancers, 5 curative cancers (2 in the ESTD group, 3 in the ESD group) were intramucosal well-differentiated cancer with negative margins and vascular invasion. The other 4 noncurative cancers are presented in Table 2. One cancer in the ESTD group presented positive vertical margin, but no residual cancer tissue from the resected specimen was found after supplemental surgery.

The mean follow-up period of endoscopy examination was 27.1 months (range 3–52 months) for the ESTD group and 27.6 months (range 5–54 months) for the ESD group. One poorly to moderately differentiated intramucosal adenocarcinoma based on the pathological results from the surgical specimen was found at 15 months after ESTD for one curative cancer at the same location, but the 3-month and 6-month endoscopic follow-ups showed negative results. In the ESD group, one intramucosal adenocarcinoma presented local recurrence at 54 months of follow-up with negative results during 4 assessments over 42 months of follow-up and then surgery was performed.

## 4. Discussion

To the best of our knowledge, this report is the first clinical study to compare the safety and efficacy of ESTD and ESD for SGLs. A case-matched controlled study was performed to minimize the differences in the patient and lesion covariates. After comparison between the two groups, ESTD was demonstrated to be faster for large SGLs than ESD. No complications were observed in the ESTD group, but one case presented MP damaged during the ESD operation. Therefore, ESTD provided a higher resection speed without increasing risk than ESD for large SGLs, which could be explained by the following advantages of ESTD. (1) The submucosal tunnel established in ESTD facilitated the lateral mucosa stretching to maintain a clear view for the operation, which could effectively avoid obstruction from the infolding of the resected mucosa after circumferential incision in conventional ESD [11]. The good visibility contributed to reducing the rates of complications and to saving time while addressing the events. In our study, just one case presented muscular damage, and the safe advantage of ESTD was not obvious. However, Huang et al. [21] demonstrated that ESTD had a lower rate of muscular injury than ESD (28.9% versus 52.6%, *P* < 0.05) in 115 patients analysis. (2) In the ESD procedure, additional submucosal injection tended to dissipate easily after circumferential incision [20]. However, the submucosal tunnel during ESTD allowed submucosal injection solutions to be mainly retained in the submucosa and thus reduced the amount and time of injection [19]. (3) The transparent cap in the front of the endoscopy and CO2 insufflation contributed to blunt dissection in the tunnel [19]. (4) ESTD enabled easier dissection close to the muscularis propria and allowed complete resection of the submucosa. This advantage had been warranted by one prospective, randomized, and comparative experimental animal study, which revealed that ESTD enabled deeper dissection than ESD according to the submucosal thickness of resected specimen [3]. This advantage also makes complete resection of lesions with ulcers or fibrosis possible [18]. (5) After the tunnel was established, the bilateral resection took advantage of traction of both sides and gravity from the high to low locations to help shorten the operation time.

There were no differences in the rates of complete, curative resection, and recurrence between the two groups, which were mainly related to the diagnosis before the operation rather than the operation itself. One gastric cancer in the ESTD group presented positive vertical margins after pathological evaluation. Then, surgery was supplemented, but no residual cancer was found in the resected specimen. One reason was that the residual cancer was missed because not all of the surgical specimen was assessed; the other reason was that the burning effect on the margin might prevent accurate pathological assessment after ESTD. During follow-up, 2 local recurrent cancers were found at 15 months and 54 months. However, the corresponding primary cancers of those recurrent cancers were curative cancers rather than noncurative cancer without supplementary treatment. Because of the limited number of the cases, the risk factors of recurrence after ESD were not analyzed in this study. Previous studies had demonstrated that the risk factors included tumor size (>30 mm) and location (upper third of the stomach) [33, 34], which were also demonstrated in this study because of all the primary cancers located at the cardia with size ranging from 50 to 60 mm. Therefore, close endoscopic follow-up after endoscopic resection was necessary for the patients with those risk factors.

Various traction methods have been devised to provide adequate tension and good visibility during the ESD procedure to improve its efficacy and safety, but as described in the review by Imaeda et al. [2], these methods have their own advantages and disadvantages due to their inherent characteristics. In this study, the novel ESTD technique was used to improve the ESD procedure without extra devices or equipment and any additional invasiveness. Recently, Miura et al. [35] reported pocket-creation method (PCM) for gastric neoplasms. The pocket can recognized the tunnel in ESTD, and these two methods have the same principles. In the procedures in ESTD, one anal incision is created before the tunnel creation, which is different from PCM. In our experience, the anal incision can serve as the endpoint of the tunnel creation and prevent excessive mucosal separation. The advantage and optimum indications of these two methods need to be further investigated.

However, there are limitations for ESTD as well. First, skilled and experienced operators are needed to perform the procedure. The creation of a submucosal tunnel is more difficult than that in the esophagus because of the gastric anatomical and physiological features, such as the large and nonstraight lumen, unfixed position, and high flexibility [23, 36]. Therefore, the operators need experience gained from ESD and other tunnel techniques to ensure the success and safety of the procedure. Second, the lesion location is a limiting factor. The cardia, lesser curvature of the gastric corpus, and greater curvature of the gastric antrum are the optimal locations to establish the submucosal tunnel based on our experience with the use of the tunnel technique in the stomach for SGLs, submucosal tumors, and gastroparesis, which also reported in previous studies [23, 36]. Although there were no lesions in the gastric antrum in this study, Choi et al. [29] reported the feasibility of ESTD for ulcerative early gastric cancer in the gastric antrum. Creating a submucosal tunnel in the other parts of the stomach is relatively difficult, time-consuming, and unsafe now. Third, ESTD is not superior to ESD for any size lesions. We assume that the tunnel section was semicircular with a minimum radius of 10 mm; therefore, the lesions with widths ≥30 mm (width = 2 × *π* × 10/2) are suitable for ESTD. However, the optimal cutoff points of lesion length and width between ESD and ESTD should be further investigated from more cases.

This study has several limitations. First, this study is one retrospective study with a limited case number in a single institution. The operators in this study have extensive experience with ESD for gastric lesions and endoscopic submucosal tunneling technique for superficial esophageal neoplasms and submucosal tumors in the esophagus and stomach. Therefore, the study results may not be generalizable. Second, the patients with SGLs performed with conventional ESD were chosen for the control group and whether ESTD was superior to other traction methods was not assessed. To provide more reliable evidence for the benefit of ESTD, we are conducting a large, multi-institutional, and prospective study.

In conclusion, this preliminary study has shown that ESTD technique is effective and safe for resecting large SGLs at suitable sites. Further prospective studies are needed to confirm the advantages of ESTD for large and ulcerative SGLs.

## Figures and Tables

**Table 1:** Baseline characteristics and treatment outcomes of the SGLs.

	ESTD (*n* = 7)	ESD (*n* = 7)	*P* value
*Baseline characteristics*			
Age (years)	63.3 ± 5.53	61.1 ± 6.96	0.59
Gender (male/female)	6/1	4/3	0.31
Lesion location			1.00
LC or PW of the cardia	5	5	
LC of lower gastric body	2	2	
Macroscopic type of lesions			0.61
0 − Is/0 − IIa/0 − IIa + IIc/0 − IIc	0/1/4/2	1/2/3/1	
Presence of ulcer/scar of lesions	0	0	1.00
*Treatment outcomes*			
Resection time (min)	69.0 ± 25.88	87.71 ± 28.61	0.01^∗^
Specimen area (mm^2^)	1181.99 ± 388.08	1166.29 ± 370.09	0.31
Resection speed (mm^2^/min)	18.86 ± 7.13	13.76 ± 3.25	0.03^∗^
En bloc resection	7	7	1.00
Complications			1.00
MP damage	0	1	
Perforation	0	0	
Postprocedural bleeding	0	0	
Pathology type			0.63
Precancerous lesion/cancer	3/4	2/5	
Complete resection	6	7	0.50
Curative resection	6	6	1.00
Endoscopic follow-up (months)	27.14 ± 16.31	27.57 ± 20.98	0.94
Recurrence	1	1	1.00

LC: lesser curvature; PW: posterior wall; MP: muscularis propria. Quantitative data are presented as mean ± standard deviation. ^∗^*P* < 0.05.

**Table 2:** The characteristics of noncurative cancers in this study.

Case number	1	2	3	4
Age (years)	69	61	69	62
Gender	Male	Male	Female	Female
Location	Cardial LC	Cardial LC	LC of LGB	Cardial LC
Specimen size (mm)	50	40	50	50
Procedure	ESTD	ESTD	ESD	ESD
Pathology				
Ulcer findings	None	None	None	None
Differentiation	tub2 > por	tub1	sig	tub1
Positive margin	None	VM (+)	None	None
Vascular invasion	Ly (+) v (+)	None	None	None
Depth	sm1	sm2	sm1	sm2
Supplemental therapy	None	Surgery^∗^	None	None
Total follow-up (months)	52 (alive)	51 (alive)	54 (alive)	40 (alive)
Endoscopic follow-up (months)	52	36	48	25
Recurrence	None	None	None	None

LGB: lower gastric body; Ly: lymphatic infiltration; v: venous infiltration; VM: vertical margin involvement; m: intramucosal cancer; sm1: invasion depth < 500 *μ*m from the lower margin of the muscularis mucosa; sm2: invasion depth ≥ 500 *μ*m; tub1: well-differentiated adenocarcinoma; tub2: moderately differentiated adenocarcinoma; por: poorly differentiated adenocarcinoma; sig: signet ring cell carcinoma. ^∗^There was no residual cancer tissue found from the resected specimen after supplemental surgery.
